# Synthesis of Bi_2_O_3_/g-C_3_N_4_ for enhanced photocatalytic CO_2_ reduction with a Z-scheme mechanism†

**DOI:** 10.1039/c9ra07485f

**Published:** 2019-11-13

**Authors:** Hao Peng, Rui-Tang Guo, He Lin, Xing-Yu Liu

**Affiliations:** School of Mechanical and Power Engineering, Shanghai Jiaotong University Shanghai 200240 China linhe@sjtu.edu.cn; College of Energy and Mechanical Engineering, Shanghai University of Electric Power Shanghai China grta@zju.edu.cn; Shanghai Engineering Research Center of Power Generation Environment Protection Shanghai P. R. China; Shanghai Institute of Pollution Control and Ecological Security Shanghai 200092 P. R. China

## Abstract

Bi_2_O_3_/g-C_3_N_4_ nanoscale composites with a Z-scheme mechanism were successfully synthesized by high temperature calcination combined with a hydrothermal method. These synthesized composites exhibited excellent photocatalytic performance, especially the 40 wt% Bi_2_O_3_/g-C_3_N_4_ composite, which produced about 1.8 times the CO yield of pure g-C_3_N_4_. The obtained products were characterized by X-ray diffraction (XRD) patterns, X-ray photoelectron spectroscopy (XPS), scanning electron microscope (SEM), transmission electron microscopy (TEM), Brunauer–Emmett–Teller (BET), UV-vis diffuse reflectance spectroscopy (UV-vis DRS) and so on. Characterization results revealed that Bi ions had well covered the surface of g-C_3_N_4_, thus restraining the recombination of electron–hole pairs and resulting in a stronger visible-light response and higher CO yield. In addition, the electron transfer process through the Z-scheme mechanism also promoted the photocatalytic activity.

## Abstract 2

Bi_2_O_3_/g-C_3_N_4_ composites were synthesized and used in photocatalytic reduction of CO_2_ with a Z-scheme mechanism.

## Graphical abstract

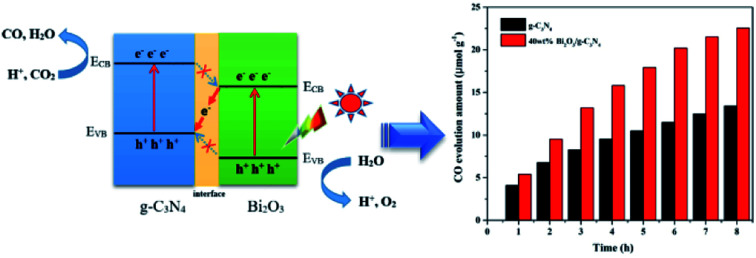

## 1. Introduction

In recent years, the continuous development of industry and the serious destruction of forest vegetation have led to difficulties in controlling CO_2_ emission, thus seriously threatening the survival and development of mankind.^1–6^ Recently, the field of CO_2_ photocatalytic reduction which translates CO_2_ into some useful substances has attracted many research teams.^7–12^ As we all know, many narrow band gap semiconductor based photocatalysts have been used in CO_2_ photocatalytic reduction.^13–15^ Among them, g-C_3_N_4_ is the most widely used one due to its suitable band gap, simple and convenient preparation and good visible light response.^16,17^ However, there are still many obvious shortcomings associated with this catalyst, such as its small range of light response, low separation rate of electron–hole pairs and unsatisfactory performance in the photocatalytic reaction. Lots of methods have been developed to improve the photocatalytic performance of g-C_3_N_4_, such as element modification,^18–20^ texture fabrication^21,22^ and heterojunction formation.^23–27^ Fabrication of a heterojunction between two semiconductors is a promising way among these methods, in which the formation of an internal electric field and the promoted separation of charge carriers effectively restrains the recombination rate of electron–hole pairs.^28^

As an important semiconductor material, Bi_2_O_3_ has been applied in many fields like electronic ceramics, optoelectronic devices, high-temperature superconductors, catalysts, and sensors due to its special physical properties and crystal structure.^29–33^ What's more, Bi_2_O_3_ also has been used as a normal photocatalyst in water splitting and photodegradation pollutant. Unfortunately, its photoreduction performance is poor owing to the low migration of photo-charges, which leads to a fast combination of photogenerated electron–hole pairs.^34–37^ For the improvement of photocatalytic ability over Bi_2_O_3_, the construction of heterojunction is considered as an effective measure to reduce the probability of electron–hole pairs recombination, like BiOCl/Bi_2_O_3_,^38^ BiVO_4_/Bi_2_O_3_ (ref. 39) and Bi_2_O_3_/TiO_2_.^40^ It is known that an internal electric field is produced after the formation of a p–n heterojunction. The photocarriers are greatly accelerated by the electric field, which effectively suppresses the reflux of photogenerated carriers and thus improves the photocatalytic performance.^41^ Due to the presence of heterojunctions, the excitation wavelength of light is also extended at the same time.^42^

From the above analysis, it can be presumed that there exists a possibility to construct a heterostructure between Bi_2_O_3_ and g-C_3_N_4_ through their mutual activation. However, the use of Bi_2_O_3_/g-C_3_N_4_ heterojunctions for CO_2_ photoreduction has not been reported. Therefore, Bi_2_O_3_/g-C_3_N_4_ compounds with different Bi_2_O_3_ contents were prepared and used in CO_2_ photocatalytic reduction in this study. It was found that the yields of Bi_2_O_3_/g-C_3_N_4_ compounds was higher than single Bi_2_O_3_ or g-C_3_N_4_. Moreover, the visible light response is also enhanced. Based on the results of all the characterization techniques, the possible promotion mechanism is also raised.

## 2. Experimental

### 2.1. Chemicals

Urea (H_2_NCONH_3_) and bismuth nitrate pentahydrate (Bi(NO_3_)_3_·5H_2_O) were purchased from Sinopharm Chemical Reagent Corp, P. R. China. All of these purchased reagents are of analytical grade and used directly.

### 2.2. Synthesis

Pure g-C_3_N_4_ was obtained by heating urea in air at 550 °C for 2 h. The Bi_2_O_3_/g-C_3_N_4_ composites with different Bi_2_O_3_ mass ratio (0, 20, 40, 60 and 80 wt%) were gained through the following procedure: g-C_3_N_4_ (0.5 g) was dissolved in 60 mL of ethylene glycol and sonicated for 30 min to yield the g-C_3_N_4_ sheet. Subsequently, a certain amount (0.104, 0.208, 0.312, 0.416 g) of Bi (NO_3_)_3_·5H_2_O and urea (1 g) were added in the solution and stirred for 1 h. The solution was then transfer to Teflon-lined autoclave (100 mL) and hydrothermally treated at 180 °C for 12 h. The obtained product was rinsed several times with deionized water and dried overnight at 80 °C. The hydrothermal product was then calcined in air at 380 °C for 2 h to get the final product. Pure Bi_2_O_3_ was obtained by the same procedure without the adjunction of g-C_3_N_4_.

### 2.3. Characterization

The crystal phase of catalyst was determined by X-ray diffraction (XRD, Bruker D8, Cu Kα radiation), and the morphology of the synthesized samples were investigated by scanning electron microscope (SEM, Phillips XL-30 FEG/NEW) and transmission electron microscope (TEM, Phillips Model CM200). The chemical elements of the compositions were analyzed by X-ray photoelectron spectroscopy (XPS, ESCALAB 250xi, USA) and Al Kα radiation sources. The Brunauer–Emmett–Teller (BET, Quantachrome Autosorb-iQ-AG instrument) pore structure and surface area were measured by N_2_ adsorption–desorption at −196 °C. UV-vis diffuse reflectance spectrum was analyzed in the range of 250–800 nm on a spectrophotometer (SHIMADZU UV-3600, Japan) using BaSO_4_ as the reflectance standard material. Photoluminescence (PL) was measured by a fluorescence spectrophotometer (Hitachi F-4600, 325 nm excitation wavelength).

### 2.4. Photoelectrochemical

Photoelectrochemical included electrochemical impedance spectroscopy (EIS) and transient photocurrent responses analysis were carried out on an electrochemical instrument (CHI 660E). A standard three-electrode system was immersed in a Na_2_SO_4_ electrolyte solution (0.5 M). The Pt and Ag/AgCl electrodes were used as counter and reference electrodes, respectively. FTO conductive glass covered by the synthesized sample was used as working electrode, and it was gained by the following method: mixture solution was consisted of Nafion (20 μL, 5%) and ethanol (1 mL). After that, 10 mg of the synthesized sample was dropped into the mixed solution and ultrasonically dispersed (2 h), then drop the slurry (0.1 mL) on 1 × 1 cm FTO glass. Therefore, the sample was well attached to the surface of the glass piece after evaporation of the ethanol. In addition, photocurrent and EIS measurements were performed under the illumination of the simulated solar light.

### 2.5. Photoactivity

The CO_2_ photoreduction experiment was performed with a reactor (500 mL) in a gas-enclosed circulation system. During the reaction, a Xenon lamp (300 W) was used as the light source. The experimental procedure was designed as follows: 50 mg sample was dispersed in 100 mL deionized water, then magnetic stirring was performed at an appropriate rotation speed. The reactor was vacuum treated and 100 kPa of high purity CO_2_ was passed into the reactor under the throttling of airflow. Then this pressure was kept for 30 min to obtain the balance of adsorption–desorption. Throughout the experiment, the temperature was maintained at 25 °C. The gas (0.15 mL) in reactor was obtained and analyzed by a gas chromatography (GC-2010 Plus, SHIMADZU, Japan) in the course of the reaction.

## 3. Results and discussion

### 3.1. SEM, XRD and TEM

**Fig. 1:**
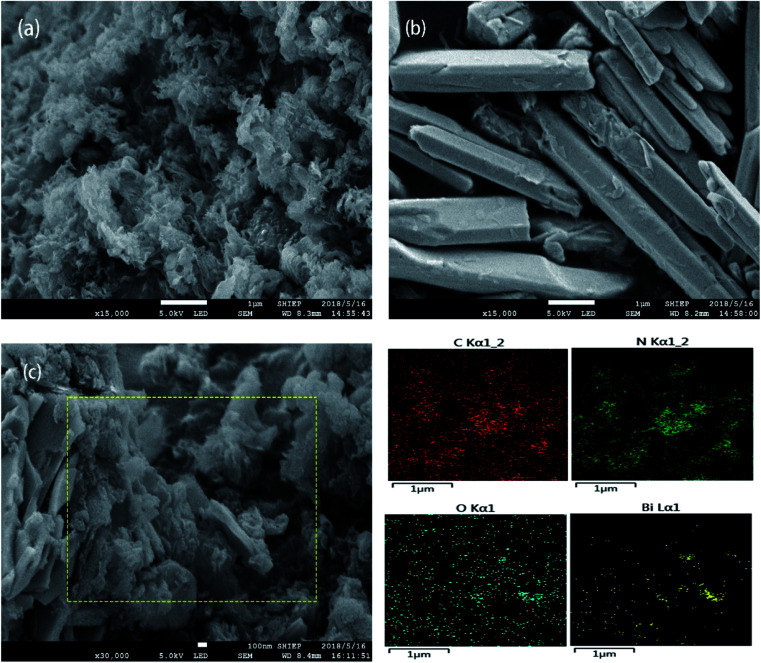
SEM of g-C_3_N_4_ (a) and Bi_2_O_3_ (b); SEM and elemental mapping of C, N, O and Bi in 40 wt% Bi_2_O_3_/g-C_3_N_4_ composite (c).

The SEM patterns of g-C_3_N_4_, Bi_2_O_3_ and Bi_2_O_3_/g-C_3_N_4_ composites are exhibited in Fig. 1. Clearly, the g-C_3_N_4_ exhibits a curled layered structure (Fig. 1a), and the Bi_2_O_3_ shows a distinct rod-like structure (Fig. 1b). Fig. 1c shows the SEM image of 40 wt% Bi_2_O_3_/g-C_3_N_4_ sample and its corresponding elemental mapping images. It could be observed from Fig. 1c that 40 wt% Bi_2_O_3_/g-C_3_N_4_ composite perfectly retains the form of Bi_2_O_3_ and g-C_3_N_4_, showing a curled sheet structure. In addition, the mapping images of 40 wt% Bi_2_O_3_/g-C_3_N_4_ composite in Fig. 1c also reveals the coexistence of C, N, O and Bi elements.

**Fig. 2:**
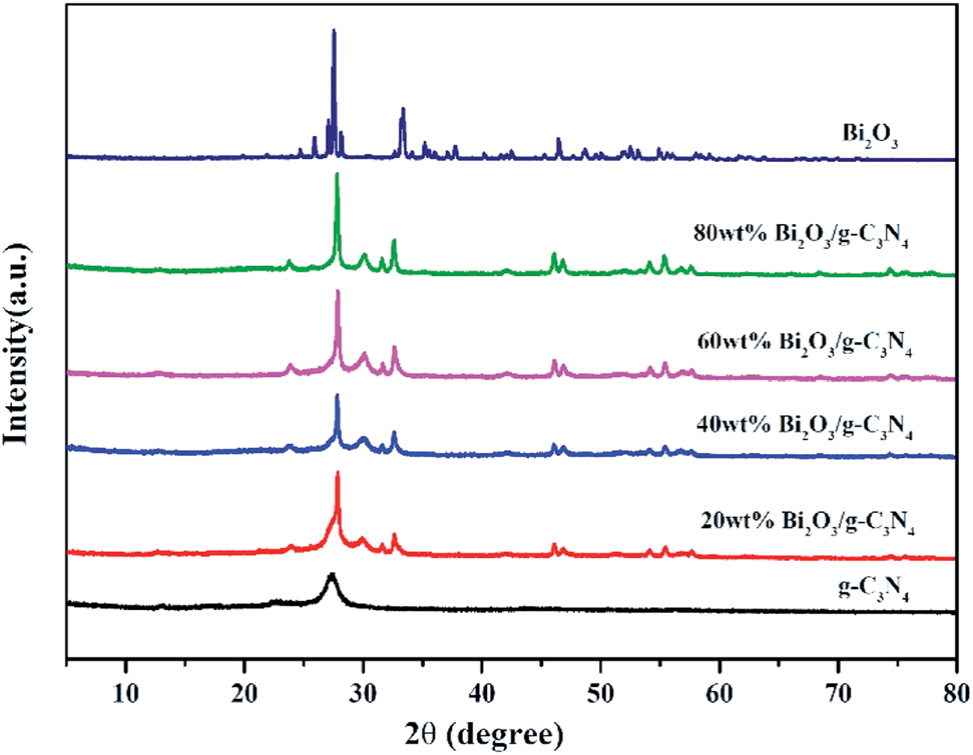
XRD of g-C_3_N_4_, Bi_2_O_3_ and Bi_2_O_3_/g-C_3_N_4_ composites.

The XRD patterns of all composites are displayed in Fig. 2. In the pattern of g-C_3_N_4_, the diffraction peak observed at 27.3° could be attributed to the (002) plane of g-C_3_N_4_ (JCPDS 87-1526). The peak at 12.7° is the (100) plane of g-C_3_N_4_, corresponding to the in-plane structure packing motif of tri-*s*-triazine units.^43^ The synthesized Bi_2_O_3_ represents six major peaks at 2*θ* = 27.8°, 31.7°, 32.6°, 46.2°, 55.4° and 74.4°, corresponding to the (201), (002), (220), (222), (421) and (423) planes respectively, which is in consistent with the β-Bi_2_O_3_ (JCPDS 65-1209). As the content of Bi_2_O_3_ in Bi_2_O_3_/g-C_3_N_4_ samples increase, the intensity of g-C_3_N_4_ peak becomes weaker. As a contrast, the peak intensity of Bi_2_O_3_ becomes stronger, especially for the diffraction peak at 27.8°, revealing the increased crystallinity of Bi_2_O_3_.

**Fig. 3:**
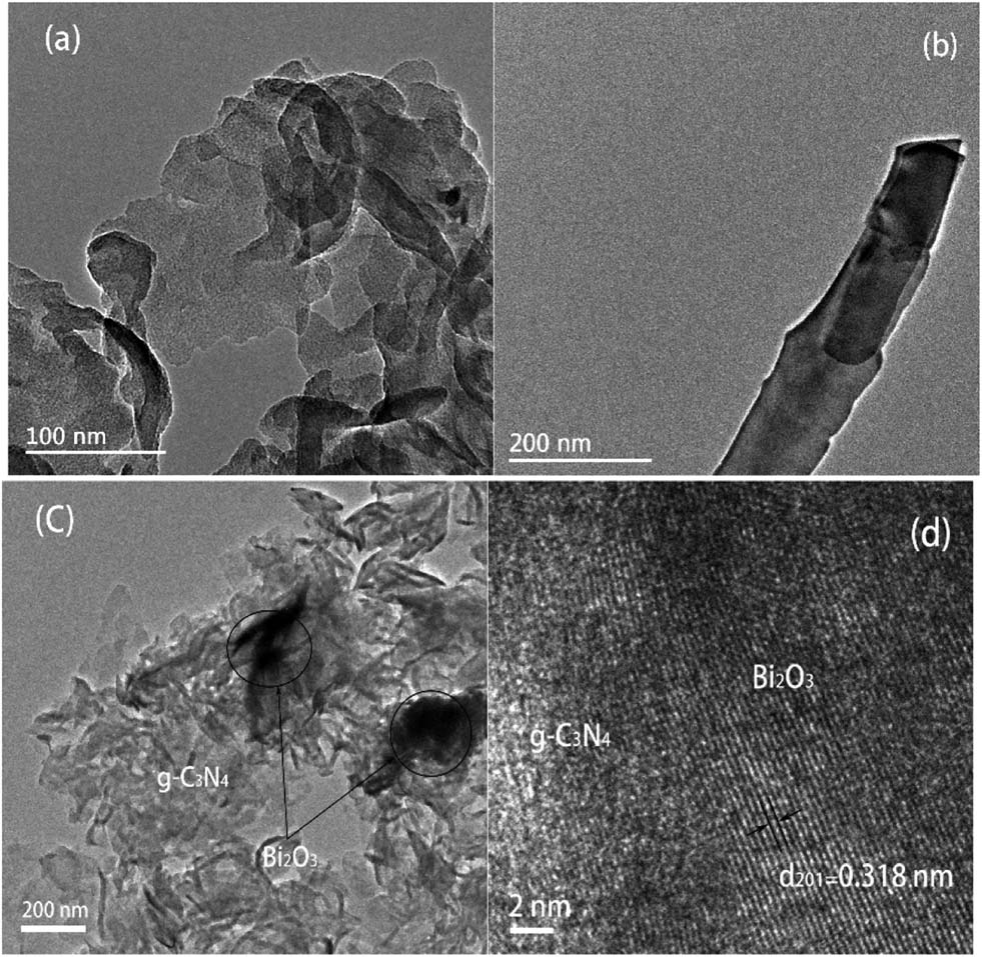
TEM image of g-C_3_N_4_ (a), Bi_2_O_3_ (b) and 40 wt% Bi_2_O_3_/g-C_3_N_4_ (c); HRTEM of 40 wt% Bi_2_O_3_/g-C_3_N_4_ (d).

The TEM images of samples are demonstrated in Fig. 3. Fig. 3a and b reveal the microscale morphology of g-C_3_N_4_ and Bi_2_O_3_, which are characterized by a curled edge and a rod-like structure respectively, as also revealed by the SEM patterns. The TEM pattern of 40 wt% Bi_2_O_3_/g-C_3_N_4_ composite shows that Bi_2_O_3_ and g-C_3_N_4_ are combined together uniformly (Fig. 3c). In addition, the 0.318 nm interplanar spacing corresponding to the (201) crystal plane of the cubic Bi_2_O_3_ is visible in the high resolution TEM image (Fig. 3d).^44^ The results show that g-C_3_N_4_ and the rod-like Bi_2_O_3_ have deeply combined together in 40 wt% Bi_2_O_3_/g-C_3_N_4_ composite.

### 3.2. XPS, BET and UV

**Fig. 4:**
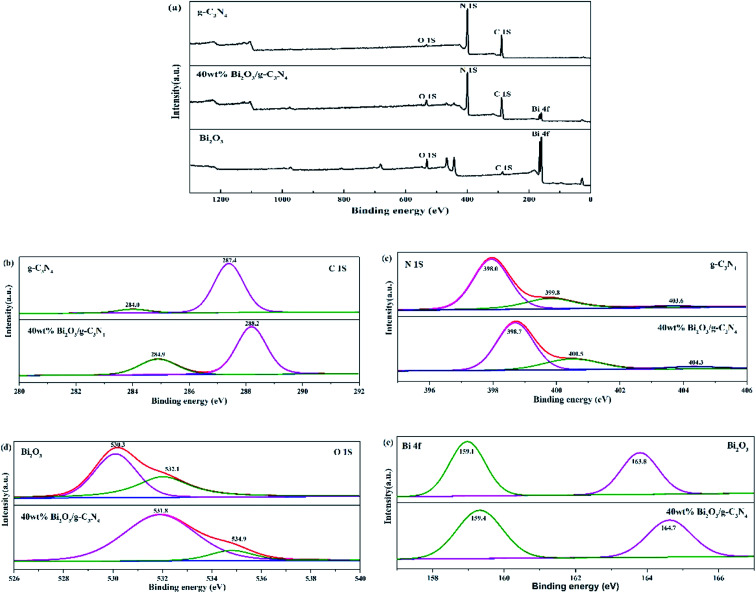
XPS of g-C_3_N_4_, Bi_2_O_3_ and 40 wt% Bi_2_O_3_/g-C_3_N_4_ (a); high-resolution C 1s (b) and N 1s (c) of g-C_3_N_4_ and 40 wt% Bi_2_O_3_/g-C_3_N_4_; high-resolution O 1s (d) and Bi 2p (e) of Bi_2_O_3_ and 40 wt% Bi_2_O_3_/g-C_3_N_4_.

The compositions of chemical elements on g-C_3_N_4_, Bi_2_O_3_ and 40 wt% Bi_2_O_3_/g-C_3_N_4_ were analyzed by XPS. As the XPS spectra show (Fig. 4a), C 1s and N 1s signals are available in g-C_3_N_4_, Bi_2_O_3_ and 40 wt% Bi_2_O_3_/g-C_3_N_4_, while Bi 4f and O 1s peaks are detected in Bi_2_O_3_ and 40 wt% Bi_2_O_3_/g-C_3_N_4_ respectively, which means that Bi_2_O_3_ is successfully doped into g-C_3_N_4_. Carbon contained in Bi_2_O_3_ sample might be caused by extraneous carbon. The corresponding detailed spectra of C 1s, N 1s, O 1s and Bi 4f are also exhibited in Fig. 4. In the C 1s spectrum of g-C_3_N_4_, two sub-bands centered at 287.4 and 284.0 eV could be observed (Fig. 4b), corresponding to the N–C=N group and the C–C bond, respectively.^45^Fig. 4c exhibits the N 1s spectra of g-C_3_N_4_ and 40 wt% Bi_2_O_3_/g-C_3_N_4_. The dominant peak at 398.0 eV could be considered as the carbon-bonded sp^2^-hybrid aromatic N (C=N–C),^46^ while the other two peaks located at 399.8 eV and 403.6 eV could be attributed to the tertiary nitrogen N–(C)_3_ groups and π excitations.^47^ The high-resolution O 1s spectrum of Bi_2_O_3_ is shown in Fig. 4d, in which the protruding peaks at 530.3 eV and 532.1 eV are derived from the Bi–O bond. Two peaks are observed in high-resolution Bi 4f spectrum of Bi_2_O_3_ (Fig. 4e): the peak of Bi 4f at 159.1 eV and 163.8 eV reveal the presence of Bi^3+^ in Bi_2_O_3_.^48,49^ The band energy shift of most peaks could be found in the spectrum of the 40 wt% Bi_2_O_3_/g-C_3_N_4_. The shift should be originated from the different electron concentrations,^50^ which are corresponding to the SEM, XRD and TEM results mentioned above.

**Fig. 5:**
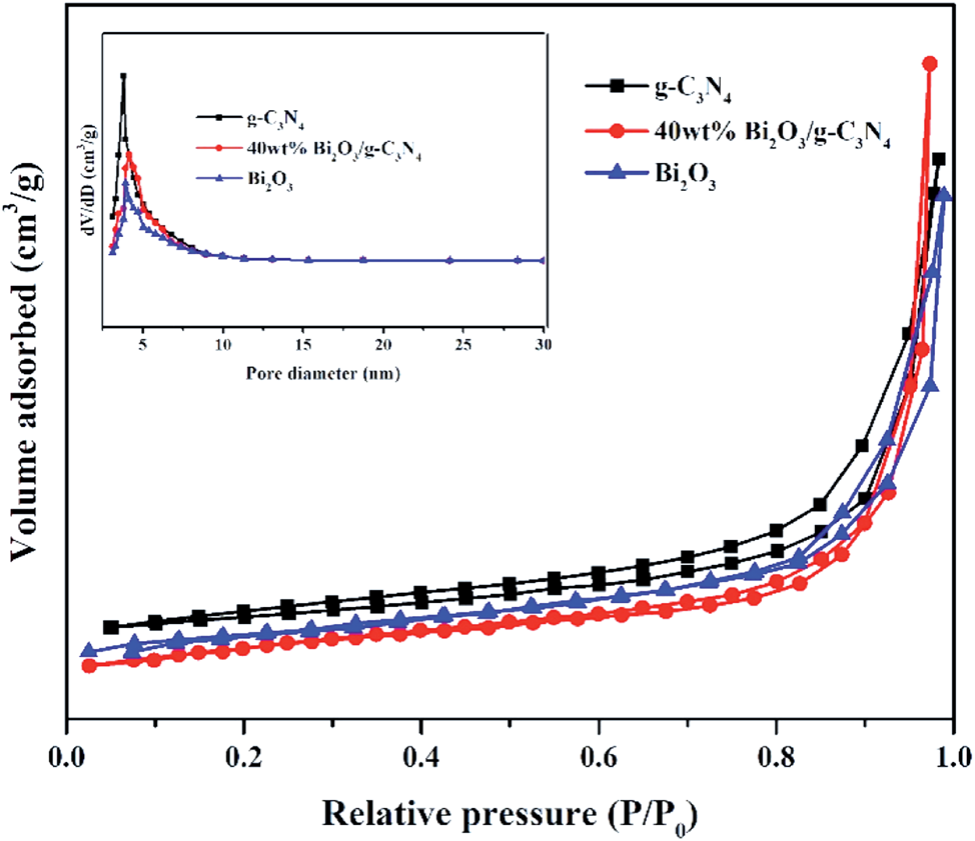
N_2_ adsorption–desorption isotherms and pore size distribution curves (inset) of g-C_3_N_4_, Bi_2_O_3_ and 40 wt% Bi_2_O_3_/g-C_3_N_4_ composite.

Nitrogen adsorption–desorption measurements were performed at −196 °C to analyze the textural features of the Bi_2_O_3_, g-C_3_N_4_ and 40 wt% Bi_2_O_3_/g-C_3_N_4_ composite. All samples show a type IV isotherm with a hysteresis loop (Fig. 5), revealing the presence of mesoporous structure into the composite. The specific surface area and average pore size of photocatalysts are listed in Table 1. Obviously, 40 wt% Bi_2_O_3_/g-C_3_N_4_ composite possesses the largest specific surface area (136.1 m^2^ g^−1^), while that for g-C_3_N_4_ and Bi_2_O_3_ are 66.7 m^2^ g^−1^ and 53.2 m^2^ g^−1^ respectively. Correspondingly, more active sites are available on 40 wt% Bi_2_O_3_/g-C_3_N_4_ composite. The BJH pore size distribution of g-C_3_N_4_, Bi_2_O_3_ and 40 wt% Bi_2_O_3_/g-C_3_N_4_ is shown in the inset of Fig. 5. Obviously, all the three samples exhibit a fairly narrow and limited pore size distribution between 3.6–4.1 nm.

**Table 1:** Textural properties of g-C_3_N_4_, Bi_2_O_3_ and Bi_2_O_3_/g-C_3_N_4_ composites

Samples	*S*_BET_ (m^2^ g^−1^)	Pore size (nm)
g-C_3_N_4_	66.7	3.839
20 wt% Bi_2_O_3_/g-C_3_N_4_	109.2	3.834
40 wt% Bi_2_O_3_/g-C_3_N_4_	136.1	3.821
60 wt% Bi_2_O_3_/g-C_3_N_4_	98.3	3.849
80 wt% Bi_2_O_3_/g-C_3_N_4_	78.2	3.815
Bi_2_O_3_	53.2	3.856

**Fig. 6:**
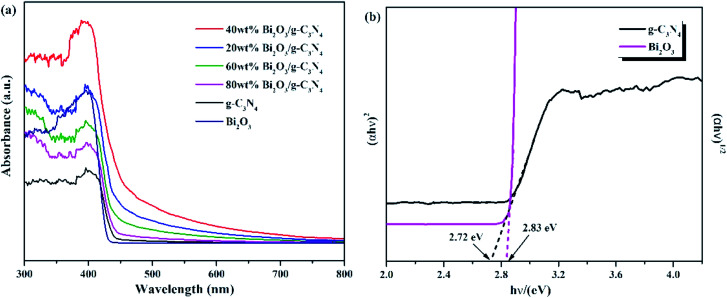
(a) UV-vis DRS for g-C_3_N_4_, Bi_2_O_3_ and Bi_2_O_3_/g-C_3_N_4_ composites. (b) (*αhν*)^1/2^ and (*αhν*)^2^*versus* energy (*hν*) for the band gap energies of g-C_3_N_4_ and Bi_2_O_3_, respectively.

The optical properties of Bi_2_O_3_, g-C_3_N_4_, and Bi_2_O_3_/g-C_3_N_4_ composites were tested by UV-vis measurement, and the relevant data were converted from the Kubelka–Munk equation. The 450 nm absorption edge of g-C_3_N_4_ could be observed in Fig. 6a, while the Bi_2_O_3_ shows a similar absorption properties. It can be found that Bi_2_O_3_/g-C_3_N_4_ samples could obtain more photons during the reaction according to the vertical coordinates, which is good to the photoreduction of CO_2_. The band gap energy of Bi_2_O_3_ and g-C_3_N_4_ could be figured out by the formula: *αhv* = *A* (*hν* − *E*_g_)^*n*/2^, where *α*, *h*, *ν*, *A* and *E*_g_ are the absorption coefficient, Planck constant, light frequency, band gap energy and constant related to the catalyst. Among them, *n* relies on optical transition type of the semiconductor (*n* = 4 for indirect transition and *n* = 1 for direct transition). For Bi_2_O_3_ and g-C_3_N_4_, the values of *n* are 1 and 4, respectively.^51–53^ According to Fig. 6b, the *E*_g_ values of Bi_2_O_3_ and g-C_3_N_4_ are counted to be 2.83 and 2.72 eV respectively.

### 3.3. Photocatalytic activity, PL and photoelectrochemical

**Fig. 7:**
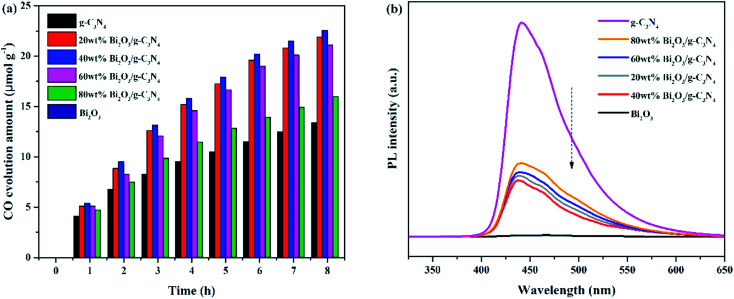
(a) Yield of CO and (b) PL spectra of all samples.

The photocatalytic activities of all samples are tested and CO was found as the main product. In addition, the control experiments were also conducted. No hydrocarbon products are tested without simulated solar light or sample, revealing that the conditions mentioned above are essential for the CO_2_ photocatalytic reduction. Fig. 7a demonstrated the amount of CO production over the photocatalysts during the light irradiation. It could be clearly seen that the pure Bi_2_O_3_ sample has not produced any CO, which means that pure Bi_2_O_3_ cannot reduce CO_2_ to CO under photocatalytic conditions, due to the fact that the CB edge of Bi_2_O_3_ is lower than the reduction potential of CO_2_/CO (−0.52 V *vs.* NHE).^54^ The yield of g-C_3_N_4_ is also not high, indicating that the photocatalytic performance of pure catalyst is not very well, which can be ascribed to the fast recombination rate of electron–hole pairs in reaction. A great enhancement of CO yield is detected in terms of the Bi_2_O_3_/g-C_3_N_4_ composites. Moreover, the photocatalytic activity enhances as the increasing Bi_2_O_3_ content in the composites, which is owing to the increased quantity of the heterojunctions. During the reaction, the highest CO yield (22.5 μmol g^−1^) is achieved on 40 wt% Bi_2_O_3_/g-C_3_N_4_, which is about 1.8 times the CO yield of g-C_3_N_4_. However, the photocatalytic performance would decline along with the excessive addition of Bi_2_O_3_, which might be aroused by the reduce of the specific surface area, and the excessive Bi_2_O_3_ might act as the recombination core of electron–hole pairs during the reaction.^55,56^ The photocatalytic stability of all composites and g-C_3_N_4_ are shown in the Fig. S1,† the yield of CO keeps stable during three experimental runs, revealing that the performance of all composites and g-C_3_N_4_ are stable during the reaction. To further probe the truth, the separation efficiency was characterized by PL. As shown in Fig. 7b, the emission peak of Bi_2_O_3_ is almost invisible in the 450–500 nm range, which may be due to a trace of surface defects or surface oxygen vacancies of Bi_2_O_3_. Compared with Bi_2_O_3_, a strong peak concentrated at 450 nm could be noticed in the g-C_3_N_4_ spectrum, which reflects the fast recombination rate of electrons–hole pairs.^57^ Different from g-C_3_N_4_, the Bi_2_O_3_/g-C_3_N_4_ composites exhibit a moderate peak intensity in the pattern, suggesting that heterojunctions are built among Bi_2_O_3_ and g-C_3_N_4_, so the holes and electrons are effectively separated. Therefore, the recombination rate is greatly reduced, which is good to enhance the activity of CO_2_ photoreduction. In addition, the 40 wt% Bi_2_O_3_/g-C_3_N_4_ has the lowest peak intensity among all the synthesized Bi_2_O_3_/g-C_3_N_4_ samples.

**Fig. 8:**
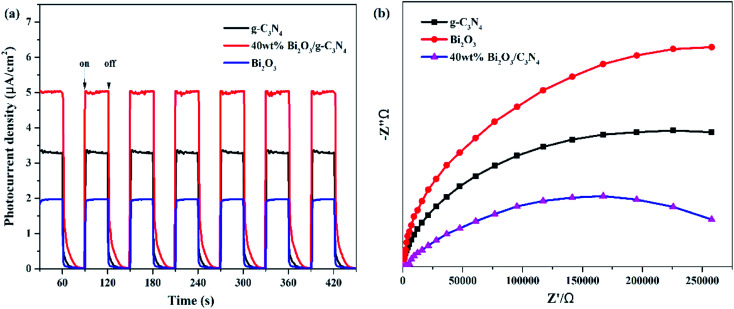
(a) Transient photocurrent density and (b) electrochemical impedance spectra of Bi_2_O_3_, g-C_3_N_4_ and 40 wt% Bi_2_O_3_/g-C_3_N_4_ composite.

In addition to PL measurements, photo-electrochemical measurements are used to further analyze the properties of photoelectrons in Bi_2_O_3_, g-C_3_N_4_ and 40 wt% Bi_2_O_3_/g-C_3_N_4_. Fig. 8a shows the photocurrent response of the samples under visible light absorption. Transient photocurrent measurements could show more evidence for the rapid electron transfer efficiency in the photocatalyst. In the light-on and light-off tests, all the photocatalysts show rapid and intense photocurrent response, indicating that the photocatalytic activity is relatively stable. Both Bi_2_O_3_ and g-C_3_N_4_ exhibit a rapidly increasing current when light is on and an abruptly decreasing signal when the light is off. This transient response behavior might be resulted from the capture and release of electrons caused by surface defects.^58^ In addition, the photocurrent intensity of 40 wt% Bi_2_O_3_/g-C_3_N_4_ heterostructured composite is 2.5 and 1.5 times the intensity of Bi_2_O_3_ and g-C_3_N_4_, respectively, indicating that 40 wt% Bi_2_O_3_/g-C_3_N_4_ has higher charge separation efficiency and better photocatalytic properties than pure catalysts. To confirm this inference, the EIS experiment was also performed. The semicircular Nynquist plots of the samples are presented in Fig. 8b. Obviously, a smaller semicircle diameter could be detected on the plot of 40 wt% Bi_2_O_3_/g-C_3_N_4_ than that of pure Bi_2_O_3_ and pure g-C_3_N_4_, indicating that the electron transfer rate is faster in 40 wt% Bi_2_O_3_/g-C_3_N_4_,^59^ which is corresponding to the previous photocatalytic activity results.

### 3.4. Mechanism

**Fig. 9:**
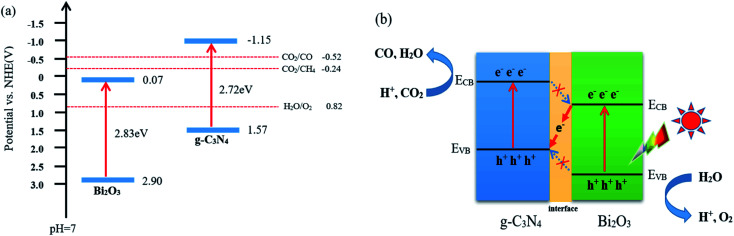
(a) Band gap energy and band positions of Bi_2_O_3_ and g-C_3_N_4_ together with CO_2_/CO, CO_2_/CH_4_ and H_2_O/O_2_ redox potentials at pH = 7 and (b) Z-type mechanism of CO_2_ photoreduction route on Bi_2_O_3_/g-C_3_N_4_ composite.

According to the characterization and CO_2_ photocatalytic reduction test results of the prepared samples, the reaction mechanism of CO_2_ reduction over Bi_2_O_3_/g-C_3_N_4_ composites were proposed. As shown in Fig. 9a, the valence band (VB) and conduction band (CB) of a semiconductor could be determined using the following equation:^60^*E*^0^_CB_ = *χ* − *E*^C^ − 1/2*E*_g_. Where *χ* is the absolute electronegativity of the semiconductor (*χ* for Bi_2_O_3_ is 5.986 eV), *E*^C^ is the energy of free electrons in the hydrogen size (4.5 eV) and *E*_g_ is the band gap of the semiconductor.^61,62^ Combined with the results in Fig. 6b, the conduction band (CB) and valence band (VB) values for the pure Bi_2_O_3_ are 0.07 eV and 2.90 eV, respectively. For g-C_3_N_4_, the VB is −1.57 eV, and the CB is 1.15 eV.^63,64^ The charge transfer in the interface can follow a double-transfer mode or a Z-scheme transfer mechanism. Double-transfer means that the CO_2_ reduction reaction would happen on the CB of Bi_2_O_3_, and the H_2_O oxidation reaction would occur on the VB of g-C_3_N_4_. In fact, the CB of Bi_2_O_3_ is lower than the reduction potential level of CO_2_/CO (−0.52 V *vs.* NHE), thus the reduction of CO_2_ would not proceed and CO would not be generated, which means that double-transfer mechanism is not suitable here to explain. Z-scheme transfer mechanism is different from the double-transfer mechanism, as shown in Fig. 9b, the photo-induced electrons on the surface of Bi_2_O_3_ can easily be transferred to g-C_3_N_4_ through the interface, the holes on VB of Bi_2_O_3_ are captured by H_2_O molecules to produce O_2_ and protons. At the same time, the CO_2_ molecules react with the electrons on the CB of g-C_3_N_4_ to generate CO and H_2_O with the participation of protons. Moreover, the formation of other products including CH_4_, H_2_, and O_2_ were also observed in the experimental process, and the charge balance table is shown in Table 2. The enhanced activity of 40 wt% Bi_2_O_3_/g-C_3_N_4_ in CO_2_ reduction is mainly due to the Z-scheme heterostructure, which effectively delays the rapid recombination of the electron–hole pairs in Bi_2_O_3_ and g-C_3_N_4_, therefore improving the photocatalytic activity.

**Table 2:** The production rate of redox products for g-C_3_N_4_ and 40 wt% Bi_2_O_3_/g-C_3_N_4_

Sample	Reduction products (μmol g^−1^ h^−1^)			Oxidation products (μmol g^−1^ h^−1^)	Ratio
	CO	CH_4_	H_2_	O_2_	e^−^ : h^+^
g-C_3_N_4_	1.68	0.38	1.11	2.42	0.89 : 1
40 wt% Bi_2_O_3_/g-C_3_N_4_	2.81	1.34	1.38	6.95	0.88 : 1

## 4. Conclusions

In general, Bi_2_O_3_/g-C_3_N_4_ nanoscale composites with heterojunction was successfully synthesized by the combination of high temperature calcination and hydrothermal method. The 40 wt% Bi_2_O_3_/g-C_3_N_4_ composite has the highest CO yield in the CO_2_ photocatalytic reduction, which is 1.8 times the yield of that g-C_3_N_4_. This obvious improvement in photoactivity is mainly due to the effective separation of electron–hole pairs and successive charge transfer through the interface. Moreover, the enhancement of specific surface area and visible light response also upgrade the photocatalytic performance of Bi_2_O_3_/g-C_3_N_4_ composite. This study gives some valuable opinion for the research of g-C_3_N_4_-related photocatalysts.

## Conflicts of interest

There are no conflicts to declare.

## Supplementary Material

RA-009-C9RA07485F-s001
