# Supplementary material for: Synthesis of Bi2O3/g-C3N4 for enhanced photocatalytic CO2 reduction with a Z-scheme mechanism
Source: RSC Adv. 2019 Nov 13;9(64):37162–70. doi: 10.1039/c9ra07485f (PMC9082309; doi:10.1039/c9ra07485f)
Supplement: RA-009-C9RA07485F-s001 [file RA-009-C9RA07485F-s001.pdf]

Electronic Supplementary Material (ESI) for RSC Advances.
This journal is © The Royal Society of Chemistry 2019

Electronic supplementary materials

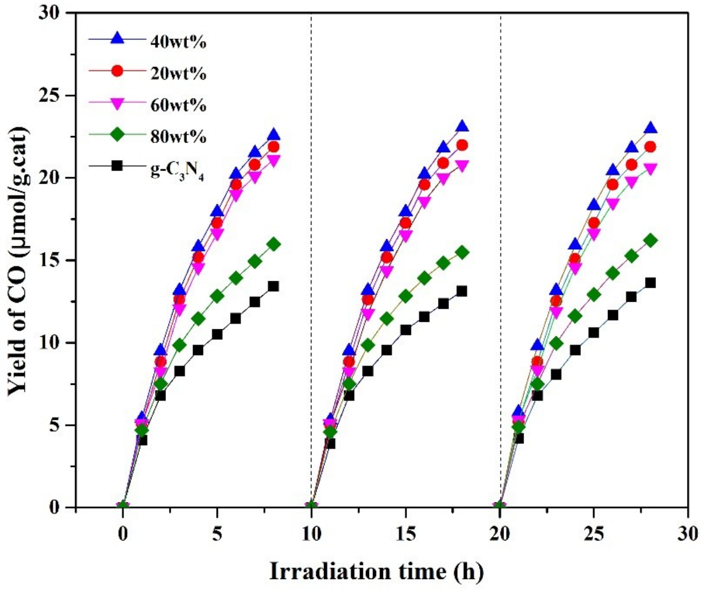

Fig. S1 Cycle characteristic diagram for all composites and pure g-C_3N_4.
